# Beliefs and Perceptions of Negative and Positive Aspects Associated With E-Cigarette Use in Young Adults of Hail City, Northwestern Saudi Arabia

**DOI:** 10.7759/cureus.94897

**Published:** 2025-10-18

**Authors:** Fahad Yousef Al-Ghaithi, Sameer Shaikh, Hussain Bassam M Qarani, Raghad Matar Alashjay, Alanoud Sultan Alshammari, Adhwaa Saud AlShammari, Raghad Nawaf Alkhamali, Turki Abdullah, Ammar Ahmed Siddiqui

**Affiliations:** 1 College of Dentistry, University of Hail, Hail, SAU; 2 Divisions of Oral Diagnosis and Oral Medicine, Department of OMFS and Diagnostic Sciences, College of Dentistry, University of Hail, Hail, SAU; 3 Department of Preventive Dental Sciences, College of Dentistry, University of Hail, Hail, SAU

**Keywords:** beliefs, e-cigarettes, hail city, perceptions, population health, saudi arabia, vaping, young adults

## Abstract

Objectives

Studies on e-cigarettes in Northwestern Saudi Arabia, particularly in the Hail Region, are limited despite their growing popularity among young adults. This study aims to investigate the beliefs and perceptions of young adults in Hail City, Northwestern Saudi Arabia, regarding the positive and negative aspects of e-cigarettes, and to examine possible associations between these perceptions and selected demographic variables such as age and marital status.

Methods

A cross-sectional, questionnaire-based study was conducted. Participants aged 18 to 34 were recruited via convenience sampling from university students and social networks. The questionnaire was distributed through social media platforms.

Results

A total of 343 participants were included. Social media (n=186, 54.2%) and friends (n=102, 29.7%) were the primary sources of e-cigarette information. Among the participants, 47.8% (n=164) who held negative perceptions about e-cigarettes expressed support for quitting vaping or discouraging its use. In contrast, 26.5% (n=91) found vaping more satisfying than smoking tobacco. A significant proportion (79.3%, n=272) believed that vaping in public harms nonsmokers, and 54.5% (n=187) thought vaping was harmful to health. Additionally, 50.4% (n=173) considered e-cigarettes as hazardous as regular cigarettes. Significant correlations were found between age, marital status, and beliefs about vaping’s benefits (p < 0.05).

Conclusion

The study highlights perceptions of e-cigarettes among youth in Hail City, revealing susceptibility to initiation despite awareness of harms. Findings of this study recommend the need for continuous monitoring, targeted interventions, and leveraging social media for education. A coordinated public health approach is crucial to prevent use, promote cessation, and protect young individuals from e-cigarettes.

## Introduction

E-cigarettes (ECs) are battery-powered devices that provide nicotine through the inhalation of vapor. People commonly refer to the act of using these e-cigarettes as "vaping." E-liquid solutions typically consist of nicotine, flavoring, and a humectant, which helps retain moisture and produces an aerosol when heated [1]. E-liquid contains a range of compounds, some of which are toxic, and the use of e-cigarettes has been associated with several health risks, including impaired lung function, worsened oral health, neurological issues, and heightened cardiovascular stress [2]. Manufacturers' and sellers' marketing strategies are an important factor in driving youth interest in e-cigarette adoption. These include promoting alluring flavors based on candies, fruits, and spices; broadcasting radio and television advertising; and sponsoring or supplying samples at youth-oriented events. E-cigarette firms also employ social media promotion as one of their marketing strategies [3]. Other common reasons for using electronic cigarettes (ECs) include curiosity, a desire to quit traditional cigarettes, and peer pressure [4]. The prevalence of electronic cigarette use among young people is increasing, leading to numerous concerns [5]. Many concerns, generally acknowledged but not fully resolved, surround the adoption of e-cigarettes by youngsters. One concern is that e-cigarettes could act as a gateway to traditional cigarettes, especially for young individuals attracted to their technological appeal or flavorings. Additional worries arise from the possibility that e-cigarettes could entice ex-smokers back into a state of nicotine addiction, impede the process of quitting among current smokers, serve as a dual-use product, or provide a means for individuals to circumvent smoking regulations [6].

Young adults' unique attributes make them a compelling demographic to research in terms of their attitudes and use of e-cigarettes. Typically, young adults are more inclined to experiment and have a greater financial capacity than teenagers to purchase e-cigarettes, which tend to be pricier than conventional cigarettes on average [7]. The risk perceptions included assessing both general and specific health hazards in terms of their potential impact. The perceived advantages included improved flavor, aroma, and safety for onlookers. The motivations for using e-cigarettes encompassed various factors such as the potential health advantages, a sense of curiosity, the desire to quit smoking, and the influence of family and friends who also use e-cigarettes. The assessment of harm perceptions and positive expectancies is critical to gaining a better understanding of the negative and positive aspects of e-cigarette use in young adults [8]. Significantly, adolescents with inaccurate conceptions of the potential risk of e-cigarettes were more vulnerable to the motivations for using e-cigarettes [9].

In Saudi Arabia, several studies indicated a significant prevalence of e-cigarette use among college students, with rates ranging from 21% to 32% [10-12]. Despite the growing body of e-cigarette research, there is a lack of evidence regarding young people's understanding and perception of e-cigarettes in Northwestern Saudi Arabia, specifically in the Hail Region. It is critical to have a thorough understanding of the information and awareness regarding the advantages and disadvantages of e-cigarettes, as their use is gaining popularity among the young population in the Hail Region.

The goal of this study is to improve understanding of young people's beliefs and perceptions about e-cigarettes. Strengthening this understanding can help in developing effective public-health strategies to encourage cessation or prevent initiation driven by curiosity or peer influence. Therefore, we conducted a comprehensive analysis of the positive and negative perceptions associated with e-cigarette use among young individuals in Hail City, Saudi Arabia. The study assessed these perceptions among young adults, regardless of whether they were current users or non-users of e-cigarettes, and aimed to examine possible associations between these perceptions and selected demographic variables such as age and marital status.

## Materials and methods

Study design and participants

An analytical cross-sectional survey was conducted among individuals residing in Hail City, Saudi Arabia, aged 18 to 34 years. The age range of 18 to 34 years was selected because it broadly corresponds to the young-adult demographic frequently used in tobacco-control research and represents the population most likely to initiate or experiment with e-cigarette use [13]. Data were collected over a six-month period, from July to December 2024. The target sample size was calculated using the Raosoft® sample-size calculator (Raosoft Inc., Seattle, WA, USA), assuming a 5% margin of error and a 95% confidence level, which resulted in a minimum requirement of 322 participants. A total of 343 participants were ultimately included in the study. A convenient sampling approach was adopted for participant recruitment, primarily involving university students, colleagues, and acquaintances who were invited to participate via social media platforms such as Snapchat and WhatsApp. A cover letter accompanying the questionnaire explained the study’s objectives and inclusion criteria. All participants provided informed consent prior to enrollment in the research study.

Questionnaire

The research instrument consisted of 20 self-administered items (Appendix 1) covering demographic characteristics, sources of information regarding e-cigarettes, and awareness of their harms, benefits, and associated perceptions. The questionnaire was originally developed in English and translated into Arabic using a forward-back translation process by bilingual experts. Any discrepancies between the versions were resolved by consensus. The questionnaire was adapted from previously published studies that explored perceptions and beliefs regarding e-cigarette use among youth [14, 15]. Items were modified to suit the local context of Hail City. Content validity was evaluated by a panel of three faculty members specializing in public health and oral medicine. Internal consistency was assessed through a pilot study involving 20 participants, yielding a Cronbach’s alpha = 0.82, indicating satisfactory reliability.

Inclusion and exclusion criteria

Participants were required to be educated adults aged 18 years or older, residing in Hail City, who could read and understand the questionnaire and had access to a smartphone. There were no gender restrictions. Participants were excluded if they were uneducated, resided outside Hail City, declined to participate, submitted incomplete or inconsistent responses, or were known to have cognitive or psychiatric impairments affecting comprehension.

Ethical considerations

The study was reviewed and approved by the Research Ethics Committee of the University of Hail (Approval No.: H-2023-373).

Data management and analysis

Data collection was anonymous. Participants completed the survey electronically without providing any identifying information. All responses were stored securely and accessed only by the research team. Data were analyzed using SPSS Version 23.0 (IBM Corp., Armonk, NY, USA). Demographic variables (age, gender, education, marital status, and nationality) were summarized as frequencies and percentages. Perceptions and beliefs regarding e-cigarette use were also expressed as frequencies and percentages. The chi-square test of independence (χ²) assessed associations between categorical variables (e.g., e-cigarette beliefs vs. age group and marital status). Test statistics, degrees of freedom (df), and exact p-values were reported. Effect sizes were calculated using Cramer’s V, interpreted as small (0.1), medium (0.3), or large (0.5) effects.

## Results

Characteristics of the study population

The study sample consisted of 343 young people residing in Hail City, Saudi Arabia. Seventy-one percent (n = 244) were female, while 29% (n = 99) were male. A total of 183 respondents (53.4%) were over 30 years old. Most participants were Saudi nationals (97.7%, n = 335) and had a bachelor’s degree or higher (69.7%, n = 239). Regarding marital status, 174 (50.7%) were married and 155 (45.2%) were single. In terms of smoking behavior, 267 (77.8%) respondents were nonsmokers, 47 (13.7%) were e-cigarette (vape) smokers, 11 (3.2%) were tobacco smokers, and 8 (2.3%) were dual users. This distribution has been included for clarity, although the study’s main objective was to explore beliefs and perceptions related to e-cigarettes among young adults, rather than to estimate the prevalence of vaping. Table 1 outlines the study participants' demographic characteristics.

**Table 1:** Study participants’ demographic details.

Category	Variables	Frequency (n)	Percent (%)
Age (years)	18-20	73	21.3%
	21-25	57	16.6%
	26-30	30	8.7%
	31-34	183	53.4%
Gender	Male	99	28.9%
	Female	244	71.1%
Nationality	Saudi	335	97.7%
	Non-Saudi	8	2.3%
Educational status	High School	62	18.1%
	Diploma	42	12.2%
	Bachelor's degree or higher	239	69.7%
Marital status	Single	155	45.2%
	Married	174	50.7%
	Separated	10	2.9%
	Widowed	4	1.2%
Are you currently a?	Nonsmoker	267	77.8%
	Ex-smoker	10	2.9%
	Smoker of electronic cigarettes (vape)	47	13.7%
	Smoker of tobacco cigarettes	11	3.2%
	Dual user (tobacco cigarettes + electronic cigarettes)	8	2.3%

The sources of knowledge and beliefs about e-cigarettes

Most respondents (54.2%, n=186) indicated that they acquired knowledge about e-cigarettes through social media. Friends also played a significant role, with 102 (29.7%) of respondents reporting that they learned about e-cigarettes from their friends. The percentage of respondents who obtained information from television (TV) and traditional print media, like magazines and newspapers, was 8 (2.3%) and 2 (0.6%), respectively. According to the public's perception of e-cigarette prevalence, a sizable majority (86%, n=295) said that e-cigarettes were commonly known or encountered in the community. The majority of respondents (74.3%, n=255) believed that it was not normal for people of their age to use e-cigarettes. A sizeable number of respondents (38.2%, n=131) indicated that friends' recommendations can be a determining factor in their choice to start vaping. The findings indicated that 164 participants (47.8%) expressed their endorsement of quitting vaping due to the negative responses about e-cigarettes they received from others. In contrast, 179 (52.2%) participants hold the belief that social responses should not serve as a deterrent for discontinuing vaping. Furthermore, 91 participants (26.5%) thought vaping to be more satisfying than smoking tobacco, while 105 participants (30.6%) perceived it as less satisfying than traditional cigarettes (Table 2).

**Table 2:** Study participants' sources of knowledge and beliefs about e-cigarettes.

Questionnaire items	Frequency (n)	Percent (%)
Where did you hear or learn about electronic cigarettes?		
Friends	102	29.7%
Social media	186	54.2%
Magazines and newspapers	2	0.6%
TV	8	2.3%
Other sources	45	13.1%
Are electronic cigarettes common in society?		
Yes	295	86.0%
No	48	14.0%
Is it normal for someone your age to use electronic cigarettes?		
Yes	88	25.7%
No	255	74.3%
What factors can influence your decision to begin vaping?		
Marketing	16	4.7%
Ease of use	61	17.8%
The influence of friends	131	38.2%
Modern appearance	35	10.2%
Attractive colors	4	1.2%
Other reasons	96	28.0%
How much satisfaction can you perceive from using vaping products compared to smoking tobacco cigarettes?		
Equal to traditional cigarettes	147	42.9%
More than traditional cigarettes	91	26.5%
Less than traditional cigarettes	105	30.6%
Do you support quitting vaping because of people's reaction to you?		
Yes	164	47.8%
No	179	52.2%

Harm perceptions of e-cigarettes

A substantial majority of respondents (79.3%, n=272) strongly agreed that vaping in public places can be harmful to non-smokers. On the other hand, a small fraction, representing 23 (6.7%) respondents, said that they did not believe vaping in public places to be harmful. Among the participants, 187 (54.5%) hold the belief that e-smoking has detrimental health effects. E-cigarettes were not considered less harmful than traditional cigarettes by 173 (50.4%) respondents. E-cigarettes were perceived as less harmful than traditional tobacco cigarettes by 67 (19.5%) respondents. Nearly half of the respondents (46.1%, n=158) did not believe that e-cigarettes are less likely to cause cancer, and 116 (33.8%) respondents expressed ambiguity regarding the relative cancer risk between e-cigarettes and traditional tobacco cigarettes. A substantial proportion of respondents believed that using e-cigarettes might cause negative problems like fatigue and exhaustion (12.2%, n=42), anger and mood swings (25.4%, n=87), lack of focus (14.9%, n=51), and anxiety and depression (6.1%, n=21). However, 142 (41.4%) respondents disagreed that using an e-cigarette causes any of these negative effects. These e-cigarettes' harmful effects would force a substantial percentage of respondents (82.5%, n=283) to give them up. Table 3 shows the harmful perceptions of e-cigarettes among study participants.

**Table 3:** Study participants' perceptions of e-cigarettes’ harmfulness.

Questionnaire items	Frequency (n)	Percent (%)
Does vaping around others cause harm to a nonsmoker?		
Yes	272	79.3%
No	23	6.7%
I don't know	48	14.0%
Do you think that vaping carries detrimental health effects?		
Yes	187	54.5%
No	156	45.5%
Do you think that electronic cigarettes are less harmful than traditional tobacco cigarettes?		
Yes	67	19.5%
No	173	50.4%
I don't know	103	30.0%
Do you think that electronic cigarettes are less likely to cause cancer than traditional tobacco cigarettes?		
Yes	69	20.1%
No	158	46.1%
I don't know	116	33.8%
Do you think that smoking e-cigarettes leads to any of the following negative effects?		
Lack of concentration and distraction	51	14.9%
Anger and mood swings	87	25.4%
Anxiety and depression	21	6.1%
Fatigue and exhaustion	42	12.2%
I cannot think of any negative effects	142	41.4%
Will you be compelled by the negative effects of e-cigarettes to quit them?		
Yes	283	82.5%
No	60	17.5%

Beliefs about positive aspects associated with e-cigarettes

According to the survey, 63 (18.4%) respondents held the belief that e-cigarettes can induce a soothing effect, alleviate stress, and enhance focus. Over half of the respondents (54.5%, n=187) perceived that e-cigarettes did not provide these benefits. Regarding the impact of e-cigarettes on quitting traditional cigarettes, 153 (44.6%) of respondents believe they did not contribute to this goal (Table 4).

**Table 4:** Study participants' beliefs about the positive aspects of e-cigarettes.

Questionnaire items	Frequency (n)	Percent (%)
Do you think that e-cigarettes calm down, relieve stress, and help focus?		
Yes	63	18.4
No	187	54.5
I don't know	93	27.1
Do you think that smoking electronic cigarettes helps in quitting smoking traditional tobacco cigarettes?		
Yes	92	26.8
No	153	44.6
I don't know	98	28.6

Relationship between various e-cigarette beliefs and study participants' age and marital status

There was a significant association between respondents’ opinions about the perceived effects of e-cigarettes on stress relief, focus, and relaxation across age groups (χ²(6) = 49.10, p < 0.001, Cramer’s V = 0.27) and across marital status groups (χ²(6) = 32.66, p < 0.001, V = 0.22), indicating small-to-moderate associations. Similarly, respondents’ belief that vaping aids in cessation of traditional tobacco use differed significantly by age (χ²(6) = 18.99, p = 0.004, V = 0.17) and by marital status (χ²(6) = 18.72, p = 0.005, V = 0.17). Satisfaction with vaping compared to smoking was also significantly associated with age (χ²(6) = 15.97, p = 0.014, V = 0.15) and marital status (χ²(6) = 16.51, p = 0.011, V = 0.16). By contrast, respondents’ perception of negative effects of e-cigarette smoking (symptoms) was not significantly associated with age (χ²(12) = 14.51, p = 0.269, V = 0.12) or with marital status (χ²(12) = 12.03, p = 0.361, V = 0.11). These non-significant results suggest that the distribution of reported symptoms was fairly similar across both age and marital status groups, indicating no meaningful differences between categories. Table 5 and Table 6 present these relationships in detail (chi-square statistics and Cramer’s V included).

**Table 5:** Relationship between various e-cigarette beliefs and study participants' age. * = statistically significant; χ² = chi-square test statistic; df = degrees of freedom; V = Cramer’s V (effect size).

Questionnaire items	Age groups (years)				Statistical analysis	
	18-20	21-25	26-30	31-34	p-value	Test Statistics (χ²(df), V)
	n (%)	n (%)	n (%)	n (%)		
Do you think that e-cigarettes calm down, relieve stress, and help focus?					<0.001*	χ²(6) = 49.10, V = 0.27
Yes	13 (17.8%)	24 (42.1%)	12 (40.0%)	14 (7.7%)		
No	36 (48.3%)	23 (40.4%)	16 (53.3%)	112 (61.2%)		
I don't know	24 (32.9%)	10 (17.5%)	2 (6.7%)	57 (31.1%)		
Do you think that smoking electronic cigarettes helps in quitting smoking traditional tobacco cigarettes?					0.004*	χ²(6) = 18.99, V = 0.17
Yes	18 (24.7%)	24 (42.1%)	14 (46.7%)	36 (19.7%)		
No	30 (41.1%)	21 (36.8%)	10 (33.3%)	92 (50.3%)		
I don't know	25 (34.2%)	12 (21.1%)	6 (20.0%)	55 (30.1%)		
Do you think you'll experience any of the following symptoms when smoking electronic cigarettes?					0.269	χ²(12) = 14.51, V = 0.12
Lack of concentration and distraction	9 (12.3%)	9 (15.8%)	1 (3.3%)	32 (17.5%)		
Anger and mood swings	16 (21.9%)	14 (24.6%)	10 (33.3%)	47 (25.7%)		
Anxiety and depression	7 (9.6%)	5 (8.8%)	3 (10.0%)	6 (3.3%)		
Fatigue and exhaustion	5 (6.8%)	6 (10.5%)	4 (13.3%)	27 (14.8%)		
I did not experience symptoms	36 (49.3%)	23 (40.4%)	12 (40.0%)	71 (38.8%)		
How much satisfaction can you perceive from using vaping products compared to smoking tobacco cigarettes?					0.014*	χ²(6) = 15.97, V = 0.15
Equal to traditional cigarettes	24 (32.9%)	21 (36.8%)	11 (36.7%)	91 (49.7%)		
More than traditional cigarettes	22 (30.1%)	22 (38.6%)	12 (40.0%)	35 (19.1%)		
Less than traditional cigarette	27 (37.0%)	14 (24.6%)	7 (23.3%)	57 (31.1%)		

**Table 6:** Relationship between various e-cigarette beliefs and study participants' marital status. * = statistically significant; χ² = chi-square test statistic; df = degrees of freedom; V = Cramer’s V (effect size).

Questionnaire items	Marital status				Statistical analysis	
	Single	Married	Separated	Widowed	p-value	Test Statistics (χ²(df), V)
	n (%)	n (%)	n (%)	n (%)		
Do you think that e-cigarettes calm down, relieve stress, and help focus?					<0.001*	χ²(6) = 32.66, V = 0.22
Yes	46 (29.7%)	13 (7.5%)	4 (40.0%)	0 (0.0%)		
No	70 (45.2%)	110 (63.2%)	5 (50.0%)	2 (50.0%)		
I don't know	39 (25.2%)	51 (29.3%)	1 (10.0%)	2 (50.0%)		
Do you think that smoking electronic cigarettes helps in quitting smoking traditional tobacco cigarettes?					0.005*	χ²(6) = 18.72, V = 0.17
Yes	51 (32.9%)	37 (21.3%)	4 (40.0%)	0 (0.0%)		
No	59 (38.1%)	90 (51.7%)	4 (40.0%)	0 (0.0%)		
I don't know	45 (29.0%)	47 (27.0%)	2 (20.0%)	4 (100.0%)		
Do you think you'll experience any of the following symptoms when smoking electronic cigarettes?					0.361	χ²(12) = 12.03, V = 0.11
Lack of concentration and distraction	21 (13.5%)	28 (16.1%)	1 (10.0%)	1 (25.0%)		
Anger and mood swings	36 (23.2%)	49 (28.2%)	1 (10.0%)	1 (25.0%)		
Anxiety and depression	14 (9.0%)	6 (3.4%)	3 (30.0%)	0 (0.0%)		
Fatigue and exhaustion	14 (9.0%)	24 (13.8%)	4 (40.0%)	1 (25.0%)		
I did not experience symptoms	70 (45.2%)	67 (38.5%)	1 (10.0%)	1 (25.0%)		
How much satisfaction can you perceive from using vaping products compared to smoking tobacco cigarettes?					0.011*	χ²(6) = 16.51, V = 0.16
Equal to traditional cigarettes	52 (33.5%)	85 (48.9%)	8 (80.0%)	2 (50.0%)		
More than traditional cigarettes	52 (33.5%)	36 (20.7%)	2 (20.0%)	1 (25.0%)		
Less than traditional cigarettes	51 (32.9%)	53 (30.5%)	0 (0.0%)	1 (25.0%)		

## Discussion

The premise of this research study is to understand the perceptions and opinions of young people regarding harm, benefits, and awareness of electronic cigarettes, also known as “e-cigarettes.” Given the growing incidence of e-cigarette use among young people, the comprehension of these ideas can influence their decision to use e-cigarettes or not. We also attempted to establish the notable correlation between the different views and opinions regarding e-cigarettes among young people when considering age and marital status. The study assessed these perceptions among young adults, regardless of whether they were current users or non-users of e-cigarettes. To our knowledge, this study is the first to examine the awareness and beliefs of young individuals about e-cigarettes in Hail, a city located in Northwestern Saudi Arabia.

Higher odds of using e-cigarettes were associated with higher popularity views and lower perceptions of harm and addiction. These connections were often stronger among nonsmokers than among current cigarette smokers [14]. In the current study, this link would be stronger because there were more nonsmokers (77.8%, n=267) than other types of smokers. The findings of this research survey revealed that social media serves as a substantial resource for acquiring knowledge regarding e-cigarettes, with more than half of the respondents (54.2%, n=186) utilizing this platform. We found that friends' role (29.7%, n=102) is another important factor in learning about e-cigarettes. However, another study from Saudi Arabia found that the majority of participants (60.4%) learned about e-cigarettes from their friends. Meanwhile, social media remained instrumental in providing information about e-cigarettes to nearly the same percentage of participants (56.3%) as in our study [15].

According to the public's perception of e-cigarette prevalence, a significant majority (86.0%, n=295) indicated that e-cigarettes were widely known or encountered in the Hail City community. The awareness of e-cigarettes among young adults in Hong Kong was 97.2%, with 16.1% having experimented with e-cigarettes (11.3% previous users; 4.8% current users). A concerning finding was that young people in Hong Kong consider e-cigarettes (and aerosols) to be less dangerous and addictive than traditional cigarettes [14].

Various elements influence young people's decisions to vape. Social factors significantly influence adolescent substance use and frequency, and vaping is no exception [16]. A US study on teenage vaping revealed that the likelihood of friends being the initial source of vaping experience was twice as high for those who had encountered e-cigarette advertisements on social media, as compared to other forms of media. Friend networks are strongly linked to social media ads and marketing for e-cigarettes. People who began using e-cigarettes due to a friend's influence were more likely to have seen e-cigarette messages on social media [17]. The influence of friends could be a decisive element to begin vaping for 38.2% of respondents (n=131) in the present study. In a quantitative online survey with a US sample of teens aged 13-18, most survey respondents (54%) tried their first e-cigarette while “hanging out with friends” [17]. At the same time, friend and family disapproval were the determinants supporting the youth’s choice not to vape [18].

Over a decade (2014-2023), a survey study among adult smokers in England revealed a notable increase in risk perceptions towards e-cigarettes. In November 2014, research indicated that 44.4% of respondents held the belief that e-cigarettes were less dangerous than cigarettes, 30.3% believed that e-cigarettes were similarly harmful, 10.8% believed that they were more harmful, and 14.5% were unaware. By June 2023, the perception of e-cigarettes as less hazardous witnessed a 40% decrease, but the perception of e-cigarettes as more hazardous had more than doubled [19]. In Barcelona, Spain, a survey evaluating the perceived risks associated with e-cigarettes among the general adult population revealed that 79.2% of individuals were aware of them. Awareness of e-cigarettes was associated with significant disparities in age, education, and smoking status. Disturbingly, the majority of Barcelona's populace holds the belief that e-cigarettes have more practical value than they do cause harm [20].

With regard to the respondents' perception of the harm of e-cigarettes in the current survey, a significant percentage of respondents (54.5%, n=187) believe that e-cigarettes have adverse health consequences. Furthermore, 50.4% of the respondents (n=173) did not perceive e-cigarettes to be less dangerous than conventional cigarettes. Only 19.5% of respondents (n=67) believe that electronic cigarettes are less harmful than traditional tobacco cigarettes. Participants in Northern England rated e-cigarettes as less dangerous than traditional cigarettes primarily due to the absence of smoke (29.8%) and a lower presence of toxins (28.9%). Among the participants who disagreed with the assertion that e-cigarettes were less dangerous than cigarettes, the primary reasons for their disagreement were a lack of reliable research on e-cigarettes (23.7%), as well as concerns about their safety (20.8%) and toxin production (18.7%) [21]. In 2016, a population-based cross-sectional survey of 4002 individuals revealed that one-fifth (20%) of Germans thought e-cigarettes were less dangerous than tobacco cigarettes [22]. Meanwhile, in the UK, 73% of the total population perceives e-cigarette consumption as less dangerous [23]. Over half of the respondents (52.4%) in a national cross-sectional study conducted in Poland expressed no concern - that is, not very concerned or totally not bothered - about the negative impact of passive smoking on their health [24]. By contrast, a noteworthy fraction of respondents (79.3%, n=272) in the present study believed that smoking e-cigarettes in public places had negative effects. An investigation conducted in Greece revealed that a 30-minute period of passive exposure to e-cigarette emissions resulted in rapid negative changes in lung function [25]. Approximately half of the respondents (46.1%, n=158) in the present survey did not think e-cigarettes were less likely to cause cancer than regular cigarettes, while 33.8% (n=116) expressed uncertainty. It is still uncertain whether e-cigarette use is associated with a long-term cancer risk. However, they have the potential to contain harmful chemicals that can disrupt DNA and result in cancer [26]. The finding of a nationally representative survey revealed that the percentage of cancer survivors who considered e-cigarettes to be equally or more detrimental than traditional cigarettes was comparable to that of non-cancer respondents (70.6% versus 68.3%). Although these findings are similar to those of people without a history of cancer, cancer survivors frequently consider e-cigarettes to be as or more hazardous than conventional cigarettes. This survey revealed a significant correlation between the perception of e-cigarette products as hazardous and their avoidance [27]. In the current study, participants judged e-cigarettes to promote fatigue and exhaustion (12.2%, n=42), irritability and mood swings (25.4%, n=87), lack of attention (14.9%, n=51), and anxiety and sadness (6.1%, n=21). According to 41.4% of respondents (n=142), e-cigarettes did not lead to any of these side effects. Due to their harmful effects, 82.5% of respondents (n=283) would stop using e-cigarettes. While health concerns may play a substantial role in motivating young e-cigarette users to quit, they are unlikely to be the only factor influencing their decision. The main factors identified in a qualitative study for the cessation of e-cigarette use included health concerns (50.9%; "I want my lungs back"), financial limitations (21.7%; "I don't have enough money to feed my addiction"), the aspiration to overcome addiction (16.0%; "I hate juuling, as it is consuming my life"), and social influences (10.1%; “it's affecting my friendships”) [28].

In terms of e-cigarettes' supposed benefits in the current study, over half of respondents (54.5%, n=187) believed they did not calm, reduce stress, or improve attention. In terms of the influence of e-cigarettes on the cessation of traditional cigarettes, 44.6% of respondents (n=153) believed they did not contribute to this objective; however, 26.8% (n=92) agreed. In a large sample (n=4,617) of Australian youths aged 12 and older, in terms of positive e-cigarette expectancies, 47% agreed or strongly agreed that using e-cigarettes relaxes people, and 45% believed that they help individuals calm down when they are stressed. Just under half of those who took the survey (47%) think that electronic cigarettes help people quit smoking, and slightly more than half (54%) believe that they can cut down on their smoking using e-cigarettes [2]. More people over 30 thought that e-cigarettes were ineffective in easing stress and smoking cessation. More married people than singles did not believe e-cigarettes help with stress or tobacco smoking cessation. Respondents' maturity and marital status would have shaped these trends in this study, affecting their perceptions of e-cigarettes. These trends align with an Australian study [2], which found that a higher proportion of young adults, compared to adolescents and adults, perceived e-cigarettes as effective smoking cessation aids and held favorable beliefs about the social and psychological benefits of e-cigarette use. Our study suggests that e-cigarettes are more likely to entice young people than adults. Consequently, young people, more than adults, are more likely to be tempted to try e-cigarettes. Overall, our research demonstrated moderate-to-considerable levels of general awareness and harm perceptions among young adults regarding e-cigarettes, as well as moderate levels of belief in their positive aspects. This trend has been encouraging when compared to previous studies that found low levels of harm perceptions [22, 23], general awareness [29], and significantly substantial levels of perceptions of the benefits of e-cigarettes [2].

Strengths and limitations

This study has several limitations. Because participants were recruited through social-media platforms, there is a potential for sampling bias, and the results may not be fully generalizable to all young adults in Hail City. The gender distribution was imbalanced, with a predominance of female respondents (71%), which may have influenced some perception-related outcomes. Although the questionnaire demonstrated satisfactory internal consistency (Cronbach’s alpha = 0.82), responses were self-reported and therefore subject to recall and social-desirability bias. In addition, the cross-sectional design precludes any inference of causal relationships between demographic variables and beliefs or perceptions regarding e-cigarette use. Furthermore, the study did not assess perceptions of e-cigarette health harms related to addiction, e-liquid toxicity, device explosions, or respiratory effects. A larger number of e-cigarette users would have provided more information about e-cigarette usage-specific beliefs.

Despite these limitations, the study has notable strengths. It is the first to explore perceptions and beliefs regarding e-cigarette use among young adults in Northwestern Saudi Arabia. Unlike most previous investigations limited to university students [10-12], this research included participants from the general population, providing a broader and more representative understanding of youth perceptions in the region. These strengths enhance the study’s contribution to the growing evidence base on e-cigarette awareness and attitudes in Saudi Arabia.

## Conclusions

Our study provides valuable insights into the perceptions and beliefs surrounding e-cigarette use among youth in Hail City, Northwestern Saudi Arabia. These results contribute to a deeper understanding of how young adults view the potential benefits and harms associated with e-cigarettes, which is essential for addressing their use in this population. The study suggests that many adolescents and young adults, even those who have never tried e-cigarettes, may be susceptible to initiation due to perceived advantages. It is also evident that young people are more inclined than older adults to experiment with vaping, suggesting that there must be continuous monitoring of e-cigarette use among this group. Despite moderate-to-high awareness of potential harms, moderate positive beliefs about e-cigarettes persist, suggesting that while youth may not adopt e-cigarettes solely due to peer pressure or curiosity, there remains a risk of initiation influenced by perceived benefits. Encouragingly, many current users may find it easier to quit, pointing to opportunities for effective intervention.

From a public health perspective, it is crucial to restrict the promotion of e-cigarettes on social media platforms, which significantly shape positive perceptions among youth. Instead, social media should be leveraged for educational purposes and to disseminate cessation resources. Addressing youth vaping through targeted guidelines and interventions in clinical settings is also vital. eHealth strategies, such as SMS-based programs and educational websites, can enhance awareness about the health risks of e-cigarettes, including respiratory hazards and chemical exposures. To achieve this, tailored and accessible digital resources should be developed to meet the needs of adolescents and young adults. A coordinated effort involving policymakers, public health agencies, parents, educators, health practitioners, and researchers is essential to discourage e-cigarette use in this vulnerable population and promote healthier choices.

## Appendices

Appendix 1: 20-Item Questionnaire

A. Demographic Features:

1. Please indicate your age (years):

☐ 18-20

☐ 21-25

☐ 26-30

☐ 31-34

2. Please indicate your gender:

☐ Male

☐ Female

3. Please indicate your nationality:

☐ Saudi

☐ Non-Saudi

4. Please indicate your educational status:

☐ High School

☐ Diploma

☐ Bachelor’s Degree or higher

5. Please indicate your marital status:

☐ Single

☐ Married

☐ Separated

☐ Widowed

6. Are you currently a:

☐ Nonsmoker

☐ Ex-smoker

☐ Smoker of electronic cigarettes (vape)

☐ Smoker of tobacco cigarettes

☐ Dual user (tobacco cigarettes + electronic cigarettes)

B. Sources of knowledge and beliefs about electronic cigarettes:

7. Where did you hear or learn about electronic cigarettes?

☐ Friends

☐ Social media

☐ Magazines and newspapers

☐ TV

☐ Other sources

8. Are electronic cigarettes common in society?

☐ Yes

☐ No

9. Is it normal for someone your age to use electronic cigarettes?

☐ Yes

☐ No

10. What factors can influence your decision to begin vaping?

☐ Marketing

☐ Ease of use

☐ The influence of friends

☐ Modern appearance

☐ Attractive colours

☐ Other reasons

11. How much satisfaction can you perceive from using vaping products compared to

smoking tobacco cigarettes?

☐ Equal to traditional cigarettes

☐ More than traditional cigarettes

☐ Less than traditional cigarettes

12. Do you support quitting vaping because of people's reaction to you?

☐ Yes

☐ No

C. Perceptions of electronic cigarettes’ harmfulness:

13. Does vaping around others cause harm to a nonsmoker?

☐ Yes

☐ No

☐ I don’t know

14. Do you think that vaping carries detrimental health effects?

☐ Yes

☐ No

15. Do you think that electronic cigarettes are less harmful than traditional tobacco

cigarettes?

☐ Yes

☐ No

☐ I don’t know

16. Do you think that electronic cigarettes are less likely to cause cancer than traditional

tobacco cigarettes?

☐ Yes

☐ No

☐ I don’t know

17. Do you think that smoking electronic cigarettes leads to any of the following negative effects?

☐ Lack of concentration

☐ Anger and mood swings

☐ Anxiety and depression

☐ Fatigue and exhaustion

☐ I cannot think of any negative effects

18. Will you be compelled by the negative effects of electronic cigarettes to quit them?

☐ Yes

☐ No

D. Beliefs about the positive aspects of e-cigarettes:

19. Do you think that electronic cigarettes calm down, relieve stress, and help focus?

☐ Yes

☐ No

☐ I don’t know

20. Do you think that smoking electronic cigarettes helps in quitting smoking traditional

tobacco cigarettes?

☐ Yes

☐ No

☐ I don’t know

## References

[1] Dawkins L, Turner J, Roberts A, Soar K (2013). 'Vaping' profiles and preferences: an online survey of electronic cigarette users. Addiction.

[2] Thoonen KA, Jongenelis MI (2023). Perceptions of e-cigarettes among Australian adolescents, young adults, and adults. Addict Behav.

[3] Cooper M, Loukas A, Harrell MB, Perry CL (2017). College students' perceptions of risk and addictiveness of e-cigarettes and cigarettes. J Am Coll Health.

[4] Cho JH (2017). The association between electronic-cigarette use and self-reported oral symptoms including cracked or broken teeth and tongue and/or inside-cheek pain among adolescents: a cross-sectional study. PLoS One.

[5] Al-Hamdani M, Hopkins DB (2023). E-cigarettes in the Middle East: the known, unknown, and what needs to be known next. Prev Med Rep.

[6] Pearson JL, Richardson A, Niaura RS, Vallone DM, Abrams DB (2012). e-Cigarette awareness, use, and harm perceptions in US adults. Am J Public Health.

[7] Wang X, Zhang X, Xu X, Gao Y (2019). Perceptions and use of electronic cigarettes among young adults in China. Tob Induc Dis.

[8] Romijnders KA, van Osch L, de Vries H, Talhout R (2018). Perceptions and reasons regarding E-cigarette use among users and non-users: a narrative literature review. Int J Environ Res Public Health.

[9] Zhao S, Li Z, Zhang L, Yu Z, Zhao X, Li Y, Zhu J (2023). The characteristics and risk factors of e-cigarette use among adolescents in Shanghai: a case-control study. Tob Induc Dis.

[10] Aqeeli AA, Makeen AM, Al Bahhawi T, Ryani MA, Bahri AA, Alqassim AY, El-Setouhy M (2022). Awareness, knowledge and perception of electronic cigarettes among undergraduate students in Jazan Region, Saudi Arabia. Health Soc Care Community.

[11] Qanash S, Alemam S, Mahdi E, Softah J, Touman AA, Alsulami A (2019). Electronic cigarette among health science students in Saudi Arabia. Ann Thorac Med.

[12] Alshanberi AM, Baljoon T, Bokhari A (2021). The prevalence of E-cigarette uses among medical students at Umm Al-Qura University; a cross-sectional study 2020. J Family Med Prim Care.

[13] Bandi P, Cahn Z, Goding Sauer A, Douglas CE, Drope J, Jemal A, Fedewa SA (2021). Trends in E-cigarette use by age group and combustible cigarette smoking histories, U.S. adults, 2014-2018. Am J Prev Med.

[14] Jiang N, Cleland CM, Wang MP, Kwong A, Lai V, Lam TH (2019). Perceptions and use of e-cigarettes among young adults in Hong Kong. BMC Public Health.

[15] Doumi R, Khaytan S, Alobaidan AS (2023). Knowledge, attitude, and practice of E-cigarettes of adolescents and adults in Saudi Arabia: a cross-sectional study. Healthcare (Basel).

[16] Skinner AT, Golonka M, Godwin J, Kwiatek S, Sweitzer M, Hoyle RH (2024). My friends made me do it: peer influences and different types of vaping in adolescence. Addict Behav.

[17] Groom AL, Vu TT, Landry RL (2021). The influence of friends on teen vaping: a mixed-methods approach. Int J Environ Res Public Health.

[18] Struik L, Christianson K, Khan S, Yang Y, Werstuik ST, Dow-Fleisner S, Ben-David S (2023). Factors that influence decision-making among youth who vape and youth who don't vape. Addict Behav Rep.

[19] Jackson SE, Tattan-Birch H, East K, Cox S, Shahab L, Brown J (2024). Trends in harm perceptions of E-cigarettes vs cigarettes among adults who smoke in England, 2014-2023. JAMA Netw Open.

[20] Martínez-Sánchez JM, Fu M, Martín-Sánchez JC, Ballbè M, Saltó E, Fernández E (2015). Perception of electronic cigarettes in the general population: does their usefulness outweigh their risks?. BMJ Open.

[21] Arshad H, Jackson SE, Kock L, Ide-Walters C, Tattan-Birch H (2023). What drives public perceptions of e-cigarettes? A mixed-methods study exploring reasons behind adults' perceptions of e-cigarettes in Northern England. Drug Alcohol Depend.

[22] Eichler M, Blettner M, Singer S (2016). The use of E-cigarettes. Dtsch Arztebl Int.

[23] Kirby T (2019). E-cigarette use in Great Britain. Lancet Respir Med.

[24] Jankowski M, Wrześniewska-Wal I, Ostrowska A, Lusawa A, Wierzba W, Pinkas J (2021). Perception of harmfulness of various tobacco products and E-cigarettes in Poland: a nationwide cross-sectional survey. Int J Environ Res Public Health.

[25] Tzortzi A, Teloniatis SI, Matiampa G (2018). Passive exposure to e-cigarette emissions: immediate respiratory effects. Tob Prev Cessat.

[26] Sahu R, Shah K, Malviya R (2023). E-cigarettes and associated health risks: an update on cancer potential. Adv Respir Med.

[27] Bjurlin MA, Basak R, Zambrano I, Schatz D, El Shahawy O, Sherman S, Matulewicz RS (2022). Perceptions of e-cigarette harm among cancer survivors: findings from a nationally representative survey. Cancer Epidemiol.

[28] Amato MS, Bottcher MM, Cha S, Jacobs MA, Pearson JL, Graham AL (2021). "It's really addictive and I'm trapped:" a qualitative analysis of the reasons for quitting vaping among treatment-seeking young people. Addict Behav.

[29] Abdel-Qader DH, Al Meslamani AZ (2021). Knowledge and beliefs of Jordanian community toward E-cigarettes: a national survey. J Community Health.

